# Human dignity in the Nazi era: implications for contemporary bioethics

**DOI:** 10.1186/1472-6939-7-2

**Published:** 2006-03-14

**Authors:** Dónal P O'Mathúna

**Affiliations:** 1 Lecturer in Health Care Ethics, School of Nursing, Dublin City University, Dublin 9, Ireland

## Abstract

**Background:**

The justification for Nazi programs involving involuntary euthanasia, forced sterilisation, eugenics and human experimentation were strongly influenced by views about human dignity. The historical development of these views should be examined today because discussions of human worth and value are integral to medical ethics and bioethics. We should learn lessons from how human dignity came to be so distorted to avoid repetition of similar distortions.

**Discussion:**

Social Darwinism was foremost amongst the philosophies impacting views of human dignity in the decades leading up to Nazi power in Germany. Charles Darwin's evolutionary theory was quickly applied to human beings and social structure. The term 'survival of the fittest' was coined and seen to be applicable to humans.

Belief in the inherent dignity of all humans was rejected by social Darwinists. Influential authors of the day proclaimed that an individual's worth and value were to be determined functionally and materialistically. The popularity of such views ideologically prepared German doctors and nurses to accept Nazi social policies promoting survival of only the fittest humans.

A historical survey reveals five general presuppositions that strongly impacted medical ethics in the Nazi era. These same five beliefs are being promoted in different ways in contemporary bioethical discourse. Ethical controversies surrounding human embryos revolve around determinations of their moral status. Economic pressures force individuals and societies to examine whether some people's lives are no longer worth living. Human dignity is again being seen as a relative trait found in certain humans, not something inherent. These views strongly impact what is taken to be acceptable within medical ethics.

**Summary:**

Five beliefs central to social Darwinism will be examined in light of their influence on current discussions in medical ethics and bioethics.

Acceptance of these during the Nazi era proved destructive to many humans. Their widespread acceptance today would similarly lead to much human death and suffering. A different ethic in needed which views human dignity as inherent to all human individuals.

## Background

The 60th anniversary of the liberation of the Nazi concentration camps has drawn attention once again to one of humanity's darker hours. Medicine and nursing in the Nazi era continue to draw attention and reflection, in part because they demand that we as humans examine who we are and why we believe we matter. The answers profoundly impact our ethics, how we treat one another.

Part of the perplexing puzzle created by the Nazi atrocities is how trained medical and nursing professionals in a modern, civilized society could have allowed what happened. Doctors and nurses, dedicated to caring for other human beings, looked on as those entrusted to their care were mistreated and killed. Even worse, some of those professionals participated in unethical and criminal activities. How could they have done this?

The search for an answer must delve into the underlying beliefs commonly held at that time. This investigation is crucial because if those beliefs prevail again we must wonder whether such unconscionable behaviour will likely follow in their path. The origins of the Nazi atrocities do not lie in concentration camps set up by a totalitarian dictatorship. They are rooted in beliefs promoted by particular social philosophies and practices that began in hospitals.

Ethics often focuses on how we decide what is ethical in a particular case or on a certain issue. Ethics must also examine where the underlying beliefs that impact those decisions come from. The Nazi programs of involuntary euthanasia, forced sterilization, eugenics and human experimentation were strongly influenced by views about human dignity current at that time. These views had been popularized in Germany and much of the Western world since the latter decades of the nineteenth century. They helped lead to the rejection of previously dominant ideas like the inherent value and dignity of all human life. Other beliefs were promoted and accepted, notions like lives unworthy to live, races unfit to reproduce, and the elimination of the unfit. Hitler was saying nothing that had not been repeatedly stated in academic and popular circles when he wrote in *Mein Kampf*:

"The state has the responsibility of declaring as unfit for reproductive purposes anyone who is obviously ill or genetically unsound ... and must carry through with this responsibility ruthlessly without respect to understanding or lack of understanding on the part of anyone" [1].

One of the more influential sources of these ideas was the 1920 book by Binding and Hoche, respected academics from medicine and law. They asked, "Is there human life which has so utterly forfeited its claim to worth that its continuation has forever lost all value both for the bearer of that life and for society?" [2]. Their answer, and that of many scientists and medical academics, was an unequivocal 'Yes.' The leading German human genetics text at the time contained much racist language, depicted Jews negatively, and advocated infanticide for disabled infants [3]. Yet it received overwhelmingly positive reviews in medical and scientific journals in many other countries, and went through five editions before 1940. One of its authors claimed it contained the essentials of the Nazi worldview and Hitler frequently used expressions directly from it. Binding and Hoche were not alone when they proclaimed:

"There was a time, now considered barbaric, in which eliminating those who were born unfit for life, or who later became so, was taken for granted. Then came the phase, continuing into the present, in which, finally, preserving every existence, no matter how worthless, stood as the highest moral value. A new age will arrive – operating with a higher morality and with great sacrifice – which will actually give up the requirements of an exaggerated humanism and overvaluation of mere existence" [4].

The "phase" to which they referred was the Christian era. Proponents of this "new age" frequently included attacks on this Western ethic because of its care and compassion for the weak and the sick. The idea that all human life had inherent dignity was replaced with the view that some human lives were not worth living and should be eliminated.

Teasing out the many philosophical influences on Hitler and Nazism is fraught with difficulties. However, a growing consensus holds that at the heart of these views was the development of social policy based on the principles of Darwinian evolution – what is known as social Darwinism. The definition of social Darwinism varies considerably, in part because social Darwinists have often held very different views on a variety of other issues. However, a useful definition is provided by Hawkins who concludes that social Darwinism is best seen as a world view consisting of five interlinked assumptions [5]. He explains the diversity of views on some ideological issues found among social Darwinists as arising from indeterminacies with the world view itself. These allow the world view to be compatible with a wide range of positions on many issues. The beliefs that Hawkins finds among social Darwinists are that:

(i) biological laws govern all of nature, including humans;

(ii) population growth puts pressure on resources that generates a struggle for existence;

(iii) physical and mental traits that confer competitive advantages in this struggle can spread throughout the population through inheritance;

(iv) selection and inheritance lead to new species appearing and others going extinct;

(v) all of the above apply to human culture, and therefore human thinking, religion, psychology, politics and ethics have evolved through natural selection.

Hawkins holds that the first four beliefs can be held without someone being a social Darwinist, but addition of the fifth belief is crucial to any definition of social Darwinism. These beliefs will be explained further as we examine the major influences on the development of social Darwinism.

While acknowledging the limitations of any definition, this paper's focus will be more on the impact of social Darwinism in Germany, especially its impact on views of human dignity. What made Germany unique was that these beliefs were imposed by a totalitarian regime [6]. At the same time it must be acknowledged that many other beliefs influenced the ideology and ethical positions ultimately promoted by Hitler and the Nazis.

The influences of social Darwinism on medical ethics must be examined carefully because Western society is currently enamoured by many of these same beliefs. They are not labelled as such, and are often promoted independently. But the ideas themselves are there and already impacting current thinking within medical ethics and bioethics.

Any attempt to draw connections between the Nazi Holocaust and contemporary bioethical debate must be done carefully. Too often connections are made that are tenuous at best, and completely wrong at worst [7]. Some claim it is impossible to draw any meaningful lessons from what was basically an "irrational lust for murder" [8]. Any mention of the Holocaust can raise so many emotions that rational discussion becomes difficult [9]. Some are offended that anything today could be compared to the Holocaust since it is viewed as the icon of absolute evil. Yet similarities do exist between some of the practices carried out by the Nazis and practices currently being debated. Some emphasise these similarities while others focus on the differences to avoid any connection. The claim is often made that "then is not now, and there is not here, and they are not us" [10]. The assumption is that we could never do what they did.

Yet they were people like us. Part of the internal anguish in examining the Holocaust comes from wondering whether we actually *could *do what they did. Some claim the Nazis were completely psychopathic. Others disagree, like Elie Wiesel who wrote that, "They did not think that what they were doing was wrong. They were convinced that what they did was good" [11]. They thought they were doing what was best for humanity, or at least for their *Volk*. Then and now, the same questions were asked. "Who shall live and who shall die? And, Who belongs to the community entitled to our protection? Then and now, the subject at hand is killing, and letting die, and helping to die, and using the dead" [12]. Then and now, similar arguments based on similar worldviews were used to justify controversial practices.

This paper will not try to assess the ethics of practices like euthanasia by making analogies between the present and the Nazi era. Rather, we will examine some of the beliefs that lay at the roots of Nazi ideology, and show parallels in contemporary bioethical thought. Before we can be too sure that we will not repeat the mistakes of Nazism, we must examine the beliefs that led them to do what they did to other humans. This will take us first to the origins of social Darwinism and how it impacted people's views on human dignity.

## Discussion

### Major influences

To understand how statements like those already quoted could have been accepted by anyone in a caring profession, we must look at some of the major ideological beliefs that influenced the development of social Darwinism. Some of these influences impacted Charles Darwin himself as he developed his theory of evolution by natural selection. Others impacted those who took Darwin's theory and applied it to social and ethical policies.

Social Darwinism is a naturalistic form of evolutionary ethics. It sought to replace the previously dominant ethical systems of the late nineteenth century: those based on transcendental ethical systems like Judeo-Christianity or philosophical systems like Immanuel Kant's deontology. The idea that nature and science could make a significant contribution to ethical and social policy represented a major shift in thinking.

#### Malthusianism

During the seventeenth and eighteenth centuries new social policies were regularly proposed to combat problems like poverty. In spite of new wealth from industrialization and colonization, poverty remained a major problem leading to implementation of various social programs [13]. Thomas Robert Malthus (1766–1834) proposed one such new and controversial approach based on biological observations that animal populations consistently outgrew whatever nourishment was available. "The cause to which I allude, is the constant tendency in all animal life to increase beyond the nourishment prepared for it.... Necessity, that imperious all-pervading law of nature, restrains them within the prescribed bounds" [14]. He concluded that poor laws were doing more harm than good and should be abandoned, leaving the poor to take responsibility for their own condition.

Malthus thus raised the possibility that continued population growth was neither natural nor necessarily good. He concluded that while giving aid to the poor appeared to be the humanitarian response, it was not the right response. His biographer noted that the avalanche of criticism he received made him the "best-abused man of the age" [15]. Charles Darwin later acknowledged that Malthus strongly influenced his thinking. More generally, though, he laid the foundation for a view of ethics based on observing biological behaviour (science), rather than on philosophy or theology.

#### Herbert Spencer

Herbert Spencer (1820–1903) is regarded as "the most influential writer of this time on general philosophy and man's place in nature. When he died he was the most famous and most popular philosopher of his age and was seen by many as a 'second Newton"' [16]. More popularly, his most famous and enduring contribution may be for coining the phrase 'survival of the fittest.' He derived this phrase from philosophical reflection, not scientific observation, six years before Darwin published *The Origin of Species *[17].

Spencer was a firm believer in the progressiveness of evolution, and taught that 'survival of the fittest' should be the rule for society. However, he became disillusioned with social policies that he believed were taking society away from this ideal. He commented in 1884 that in spite of the "truth" of 'survival of the fittest' being "recognized by most cultivated people ... now more than ever, in the history of the world, are they doing all they can to further the survival of the unfittest!" [18].

Spencer rejected the idea of caring for the poor and sick. "The poverty of the incapable, the distresses that come upon the imprudent, the starvation of the idle, and those shoulderings aside of the weak by the strong ... are the decrees of a large, far-seeing benevolence" [19]. Spencer became an advocate for policies that would help only the fittest to survive. Spencer's views differed radically from ideas prevalent in the health care professions that the sick, disabled, and the weak ought to be cared for because of their weakness and vulnerability [20]. Such beliefs were based on ideas like the inherent dignity of all humans, the sanctity of human life and the notion that all humans were entitled to certain rights. These views of human dignity were violently rocked by the publication of Darwin's scientific treatise.

#### Natural selection

Charles Darwin (1809–1882) proposed in *The Origin of Species *that all biological variation could be explained on the basis of natural selection [21]. Later scientific work connected the source of natural variation with genetic mutations that randomly appear in species' genes. Those variations that improve individuals' survivability, and their ability to leave offspring, will be found in larger proportions of subsequent populations. Useful, random changes are thus naturally selected because they help the organism adapt better and survive longer. Thus, Darwin boldly claimed to explain the origin of all species by natural mechanisms – not special creation. He himself claimed that this was "the doctrine of Malthus applied with manifold force to the whole animal and vegetable kingdoms" [22].

The impact of Darwin's work has been monumental. One authority claims that, "Next to the Bible no work has been quite as influential, in virtually every aspect of human thought, as *The Origin of Species*" [23]. The evolutionary philosopher, George Gaylord Simpson, proposed one reason for this, which applies most directly to our topic here. "The Darwinian revolution changed the most crucial element in man's world – his concept of himself" [24]. Simpson later claimed that all attempts prior to 1859 to answer the question 'What is humanity?' are worthless and ought to be ignored completely [25].

Darwin was initially reluctant to apply natural selection to humans. His early publications avoided addressing the issue, but left the door open for others to see the implications. In *The Origin*, he wrote:

"It may be difficult, but we ought to admire the savage instinctive hatred of the queen-bee, which urges her instantly to destroy the young queens her daughters as soon as born, or to perish herself in the combat; for undoubtedly this is for the good of the community; and maternal love and maternal hatred, though the latter fortunately is most rare, is all the same to the inexorable principle of natural selection" [26].

By 1871 Darwin could no longer hide his views on human evolution and he published *The Descent of Man*. However, his notebooks from 1838 contain many ruminations on the implications of his work for humans and in particular for ethics and morality [27]. *The Descent *claimed that in primitive tribes "the weak in body or mind are soon eliminated" in contrast to "civilised men" who "do our utmost to check the process of elimination; we build asylums for the imbecile, the maimed, and the sick; we institute poor-laws; and our medical men exert their utmost skill to save the life of every one to the last moment" [28]. He saw this as "highly injurious to the race of man." In spite of this, he predicted that, "At some future period, not very distant as measured by centuries, the civilised races of man will almost certainly exterminate, and replace the savage races throughout the world" [29]. His ideas were quickly accepted, though Darwin noted that they were most rapidly welcomed and promoted in Germany. Before examining this, we must briefly explore another aspect of evolutionary theory.

#### Heredity

While natural selection was accepted as the method by which changes were selected, the mechanism by which the changes initially occurred was unclear. DNA and genetics were unheard of then. Although initially sceptical of the Theory of Acquired Characteristics proposed by Jean Baptiste de Lamarck (1744–1829), Darwin gradually came to accept the idea [30]. This view held that organisms adapted to the pressures of their environment and would then pass on those acquired characteristics to their off-spring. Giraffes can be used in a simplified example. When feeding, giraffes stretch their necks to reach higher and higher leaves. By continuously doing this, their necks might get longer. According to Lamarck's theory, the longer-necked giraffes would eat more, live longer, and have more offspring. Gradually, then, giraffes would evolve into animals with longer necks.

Lamarck's theory was applied beyond zoology. It gave hope to social reformers that education and other social policies could lead to improvements in humans that would be passed on to future generations. However, the theory received a significant blow when August Weismann (1834–1914), a German biologist, published the results of experiments with mice in 1888 [31]. Weismann cut the tails off nine hundred mice over six generations – yet all the offspring grew tails. Changes made in one generation were not being passed on to the next generation. Social reformers who had accepted Lamarck's theory saw implications for their policies. Now, it seemed, some people were destined to be a certain way, and little or nothing could be done about it. Weismann used the term 'germ' to describe the earliest physical manifestation of an individual, much as we might use the term 'genome' to refer to hereditary material. He claimed:

"We cannot by excessive feeding make a giant out of the germ destined to form a dwarf; ... or the brain of a destined fool into that of a Leibnitz or a Kant, by means of much thinking. ... Hence natural selection, in destroying the least fitted individuals, destroys those which from the germ were feebly disposed" [32].

Natural selection and evolution appeared to support claims that humans change in the same way as other animals. Even if social policies impacted one generation, the evidence against Lamarck's theory implied that these changes would not be inherited. Changing those who were weak and unfit would not apparently provide a lasting solution. The logical, though controversial, next step was to propose eliminating weakness and degeneracy by preventing those with certain traits from passing them on to later generations.

#### Eugenics

The discovery that variations occur randomly and are then transmitted genetically raised the possibility that change could be directed. Francis Galton (1822–1911) coined the term eugenics to describe "the study of agencies under social control that may improve or impair the racial qualities of future generations either physically or mentally" [33]. At this time in history, the Western world held to a profound belief in progress, and evolution was seen as something that could be used to further human progress. A widely disseminated poster represented eugenics as a tree, with the caption, "Eugenics is the self-direction of human evolution."

Many at this time also accepted that all human traits were genetically determined. Leading academics from scientific and other fields held that "qualities such as intelligence, mental illness, work ethic, criminality and poverty were inherited" [34]. The way to improve society was thus to encourage those with 'good genes' to reproduce and discourage those with 'bad genes' from having children. In the US, this led to laws restricting immigration and forcing sterilization on certain people. Many European countries considered similar laws, and some implemented them. In Germany, calls for eugenics soon got entangled with ideas like racial hygiene, anti-Semitism, and Nazism. Rather than just preventing the unfit from reproducing, the policy became one of eliminating the unfit. No one author better exemplifies the application of these principles than Ernst Haeckel (1834–1919).

#### Ernst Haeckel

Haeckel was a scientist who went on to become a prolific popular author. He wrote the three most popular non-fiction books in Germany at the turn of the century [35]. Haeckel's views on ethics and morality featured prominently in all these books, working out the implications of natural selection for human society. He congratulated Darwin on his seventieth birthday for having "shown man his true place in nature and thereby overthrowing the anthropocentric fable" [36]. This fable referred to Christian teaching that humans were specially created and were therefore entitled to special protections, the so-called sanctity of human life. Foremost in Haeckel's mind was the belief that Darwinism made God, and in particular the God of Judeo-Christianity, superfluous.

Haeckel's ethics and social policies started from the premise that human worth was not inherent, but dependant on fitness and potential contribution to society. Human progress is based on "the struggle for existence and natural selection" which Haeckel viewed as natural laws. For him, then, "politics is applied biology." Haeckel's social Darwinism led him to propose some radically different claims about what was ethical regarding humans. As early as 1870 he stated:

"If someone would dare to make the suggestion, according to the example of the Spartans and Redskins, to kill immediately after birth the miserable and infirm children, to whom can be prophesied with assurance a sickly life, instead of preserving them to their own harm and the detriment of the whole community, our whole so-called 'humane civilization' would erupt in a cry of indignation" [37].

He went on to make clear that he advocated infanticide, and also abortion, assisted suicide, and the involuntary killing of the mentally ill. He later added to this group, lepers, cancer patients and those with incurable diseases whose lives were "totally worthless" and a burden on society. Underlying all these proposals was his belief stated in 1864 that, "Personal individual existence appears to me so horribly miserable, petty, and worthless, that I see it as intended for nothing but for destruction" [38].

### The path to Nazi medicine

Other factors contributed to the situation in Germany that led to the acceptance of Nazi policies. Economic pressures on the healthcare system were severe in the early twentieth century. For example, between 1885 and 1900 the number of people in the mental asylums of the state of Prussia increased by 429 percent while the general Prussian population increased by only 48 percent [39]. A prominent science journal held a competition in 1911 (with a considerable cash prize) for the best essay on the topic, "What do the bad racial elements cost the state and society?" [40]. The winning essay examined the costs of institutionalizing "inferior" people in Hamburg, and was later used by an anatomy professor at the University of Vienna to support his claim that, "As cruel as it may sound, it must be said, that the continuous ever-increasing *support of these negative variants is incorrect from the standpoint of human economy and eugenically false*" [41]. Only when the economic pressures were combined with social Darwinism's view of human dignity did the elimination of the weak and unfit become an acceptable option.

Under such influences, medicine and nursing changed dramatically in early twentieth-century Germany. In addition to economic pressures and eugenics, social Darwinism's prioritization of race over individual impacted the day-to-day activities of doctors and nurses. Warren Reich has demonstrated how the notion of care itself was transformed during this time. Popular reformers of the healthcare system advocated moving the traditional emphasis on care as concern for sick or weak individuals to a view of care as preservation of the health of those who had the most to contribute to society. This was based on "the meaninglessness of the individual in the larger biological picture" [42]. This transformed medical and nursing attitudes towards their practice and the meaning given to the duty to care. Thus, "Nursing care was not to be given to the weak. Nurses were cautioned against trying to show false mercy to uselessly sick people; and, in fact, nurses were taught that taking care of 'useless' people did harm to the nurses themselves" [43]. All based on the ideology of social Darwinism.

German medical academics combined such principles with racist ideas. Alfred Ploetz co-founded the German Society for Racial Hygiene in 1904. A sociologist with whom he worked closely stated that the highest moral principle is, "Everything that promotes the increased reproduction of the more fit racial elements, even if [it is] at the expense of the unfit" [44]. Ploetz attacked Christian morality as too focused on love and sacrifice. Wilhelm Schallmayer rejected Christianity because of its concern for the weak and vulnerable which counteracted natural selection. Instead, Schallmayer held that the first state to adopt evolutionary ethics would prevail over all others in the struggle for existence [45]. To the notion of the survival of the fittest individual within a society was now added the notion of the struggle of one society against the other – so that the fittest race would survive. Extermination and war then became moral goods to eliminate the weak.

What remained was for someone to put social Darwinism into practice. One historian notes that by the time Hitler was living in Vienna in the 1920s, the press was "saturated with racist social Darwinism" [46]. How exactly Hitler picked up these ideas is uncertain, but his writings reveal his acceptance of the social Darwinist view of human dignity. He justified the strong asserting their will over the weak by claiming "it's the law of nature" [47]. Hitler, like Haeckel, turned to Sparta as an exemplary society that implemented the type of social policy he favoured. Hitler put it this way:

"Sparta must be regarded as the first folkish state. The exposure of the sick, weak, deformed children, in short their destruction, was more decent and in truth a thousand times more humane than the wretched insanity of our day which preserves the most pathological subject" [48].

### Five beliefs and their impact on bioethics

During the decades following the publication of *The Origin of Species*, a set of beliefs emerged that radically changed how people viewed human dignity. This dramatically altered what was held to be right and wrong in the way humans treated other humans. It profoundly impacted what doctors and nurses viewed as ethical. The particular beliefs varied somewhat among social Darwinists, but were united in being seen as implications of biological evolution for ethics. The following list brings together themes threaded throughout the writings of social Darwinists and presents them as five beliefs directly impacting views of human dignity and ethics [49].

1. The nature of ethics is relativistic, not universal. Ethics and morality emerged and evolved as humans and human society developed and changed. Thus, ethics must change as the environment changes. Traditional beliefs must change, including those about human dignity.

2. The distinction between humans and other animals is blurred because humans gradually evolved from other species, as opposed to having been specially created and thus endowed with unique dignity.

3. Human inequality exists in nature and leads to gradations of fitness. Race and physical and mental abilities become determinants of human dignity – as opposed to all humans having inherent dignity.

4. At the lower end of the spectrum, some lives have so little value (or quality) that they become 'lives not worth living.'

5. Natural selection shows that survival of the fittest is a law of nature. Therefore, policies that bring about the death of those not fit for survival become ethical. At the very least, it becomes ethical not to help those humans deemed to be less fit.

These beliefs are important to recognize because similar ideas are increasingly being advocated today by some within bioethics and society more generally. While not typically presented as a revival of social Darwinism, these beliefs are offered piece-by-piece in certain approaches to ethical dilemmas. Some examples will be given to support this view.

As society becomes increasingly enamoured by these views, all involved in healthcare should examine the influence and validity of these beliefs carefully. Hitler believed that his racist and eugenic practices were ethical and could be defended based on social Darwinist presuppositions. It seems unlikely that similar policies will be forced upon Western society by totalitarian regimes. But little by little, a society that accepts social Darwinist presuppositions will come to accept eugenic practices and policies. Widespread individual acceptance of such views could just as likely lead to widespread discrimination and maltreatment of those deemed unfit to survive.

#### 1. The nature of ethics

The atrocities of Nazi Germany put evolutionary ethics into bad repute for some decades. Recently, though, there has been renewed interest in the idea [50]. Pulitzer-Prize-winning author and Harvard biology professor, E. O. Wilson, has been called "Darwin's natural heir" and his book *Sociobiology: A New Synthesis*, a Darwinian manifesto [51]. Wilson's sociobiology is based on the premise that all human behaviour can be explained within an evolutionary framework. "Morality, or more strictly our belief in morality, is merely an adaptation put in place to further our reproductive ends. ... Ethics as we understand it is an illusion fobbed off on us by our genes to get us to cooperate. It is without external grounding" [52]. John Fletcher, a bioethicist whose writings on gene therapy have been very influential, claims that religion also is "an evolutionary program fulfilling a very important function: to make you aware that you're part of the whole" [53].

Wilson claims ethics can be divided into two completely different approaches. "Centuries of debate on the origin of ethics come down to this: Either ethical principles, such as justice and human rights, are independent of human experience, or they are human inventions" [54]. If ethics arises biologically, as naturalistic evolution holds, then it is inherently changeable, as are views on human dignity.

With the denial of transcendent ethical principles, calculations of physical risks and benefits become the way to make ethical decisions. James Watson shared the Nobel Prize for his role in discovering the structure of DNA and was the first director of the Human Genome Project. He rejects the notion of individual rights claiming, "This word *right *gets very dangerous. We have women's rights, children's rights; it goes on forever" [55]. In the same discussion Watson rejects appeals to public consensus to determine which genetic experiments should be permitted on humans. "I'm afraid of asking people what they think. Don't ask Congress to approve it. Just ask them for money to help their constituents....Frankly, [the public] would care much more about having their relatives not sick than they do about ethics and principles."

In the same paragraph, Watson praises the American system for "not having cowboys doing things they shouldn't." He dismisses ethical principles, yet also appeals to one. "We should treat other people in a way that maximises the common good of the human species." For him, that includes manipulating the human genome in experimental ways without any regulation, unless "there's a terrible misuse and people are dying."

The ethics promoted by Watson arise out of his Darwinian worldview where the guiding principle is the further evolution of the human species. The focus is on genetic change, since, as Watson stated before a US congressional committee, "We used to think that our fate was in our stars. Now we know, in large measure, our fate is in our genes" [56]. Without individual rights, public consensus or transcendent ethical principles, individuals will not be protected against what might promote the good of the majority. For Watson and bioethicists from the same world view, human society needs to eliminate defective genes, which justifies embryo selection, abortion, and infanticide. Gene therapy, both somatic and germ-line, is also accepted as a way to improve the human genome. At the same time, ethics is reduced to one more tool that facilitates evolutionary progress through the elimination of the weak. "For a naturalistic approach, in the last analysis, ethics is a product of a long evolutionary process" [57].

#### 2. Human distinctiveness

Peter Singer is a bioethicist who, probably more than any other author, has drawn out the ethical implications arising from claims that humans are not distinct from other animals. He does so within a utilitarian perspective, but also stresses the evolutionary nature of humanity. Singer has recently promoted the idea that those on the political left should turn to Darwinism in response to the collapse of communism [58]. In his book, *A Darwinian Left*, he develops his argument that Darwinism can be used to support the social and political views typically held by the Left [59].

Singer is better known for his controversial bioethical positions and his promotion of animal rights. He uses the term "speciesism" to criticise claims that humans have any more rights than other species. "In other words, I am urging that we extend to other species the basic principle of equality that most of us recognize should be extended to all members of our own species" [60]. Another author has put it this way:

"Most human thinkers regard the chimp as a malformed, irrelevant oddity while seeing themselves as stepping-stones to the Almighty. To an evolutionist this cannot be so. There exists no objective basis on which to elevate one species above another. Chimp and human, lizard and fungus, we have all evolved over some three billion years by a process known as natural selection" [61].

Viewing humans and animals as equals might somehow raise the ethical standards by which all species are treated. However, the early twentieth century social Darwinists used this idea to treat some humans in ways that previously had been reserved for animals. Rather than elevating the status of all species, they rejected belief in the inherent dignity of all human life and justified the killing of innocent humans believed to be of lower status than some animals. The same agenda is apparent in the title of Singer's previously cited book, *Unsanctifying Human Life*. Rejection of the idea that human life is somehow 'set apart' from other species is found throughout Darwinian literature. Just as earlier social Darwinists and Nazis advocated killing off weak and unfit humans, Singer similarly justifies infanticide and euthanasia, and claims that some animals have a higher moral standing than certain humans.

#### 3. Human gradation

Having eliminated any special status for humans, Singer and those who hold similar views, must use some criteria to grade humans lives. This, we saw with the social Darwinists and Nazis, becomes arbitrary. Today, we see similar decisions being made about human lives, especially where ethical dilemmas abound at the beginning and ending of life. One of the infertility experts attempting to clone a human baby responded to concerns about adverse effects in resulting babies by replying, "We can grade embryos. We can do genetic screening. We can do quality control" [62].

Some claim that such quality control is different when applied to human embryos. However, when accepted for some members of the human species, it can quickly spread. 'Quality control' for humans was accepted at the beginning of the twentieth century, and it is already expanding again at the beginning of the twenty-first century. Professor John Harris is a member of the British Medical Association's ethics committee and in 2004 he declared:

"There is a very widespread and accepted practice of infanticide in most countries....What do we really think is different between newborns and late foetuses? There is no obvious reason why one should think differently, from an ethical point of view, about a foetus when it's outside the womb rather than when it's inside the womb" [63].

Harris used the same relativistic argument used by Haeckel and Hitler: because other cultures practice infanticide, we ought to be open to the practice also. Daniel Dennett comes to the same conclusion in his book about how Darwinism puts "our most cherished visions of life on a new foundation" [64]. He claims that Darwinism makes it absolutely clear that there is no way to determine when a human life begins or ends. Instead, he believes "we all do share the intuition that there are gradations of value in the ending of human lives." Since nature allows many to die through miscarriages, he suggests that steps be taken to ensure that a severely deformed infant "dies as quickly and painlessly as possible." The implication is that once we have graded human lives, some will not be worth living.

#### 4. Life not worth living

The notion of human lives not worth living was central to the ideological changes at the beginning of the twentieth century. Law-suits over wrongful life claims reflect a similar view. The idea is that some human lives are so debilitated or painful that it was wrong to allow them to come into existence. A group of four internationally renowned bioethicists defend genetic tests and therapies that would lead to the prevention of certain births. They advocate a distinction between "a worthwhile life" and "a life not worth living" [65]. The latter "is a life that, from the perspective of the person whose life it is, is so burdensome and/or without compensating benefits as to make death preferable." They then argue that since persons can determine for themselves when their future lives are not worth living, it is ethical to make such determinations pre-natally and terminate embryos or foetuses whose future lives would not be worth living.

The search for ways to grade human lives is often conducted in terms of human personhood. In this approach, those viewed as persons are granted rights and protections. Those humans viewed as non-persons need not be given the same rights or protections and may therefore be killed. Personhood, within a naturalistic evolutionary perspective, is typically determined on the basis of physical characteristics. For example, Walter Glannon notes that, "A person begins to exist when the fetal stage of the organism develops the structure and function of the brain necessary to generate and support consciousness and mental life" [66]. He goes on to argue that "testing and selective termination of genetically defective embryos is the only medically and morally defensible way to prevent the existence of people with severe disability, pain and suffering that make their lives not worth living for them on the whole" and that "we are morally required to prevent the existence of people with lives that on balance are not worth living."

Debate over personhood becomes ethically problematic when combined with the notion of lives not worth living. Historically, viewing some humans as 'non-persons' has always been used "as a permissive notion that takes the moral heat off certain quandaries raised by modern medicine" [67]. Hence, Singer declares that, "Killing a defective infant is not morally equivalent to killing a person. Very often it is not wrong at all" [68]. Singer suggests that newborns can be viewed as non-persons if they are not wanted. "Thousands of years of lip-service to the Christian ethic have not succeeded in suppressing entirely the earlier ethical attitude that newborn infants, especially if unwanted, are not yet full members of the moral community" [69].

The similarities with the claims and proposals of early social Darwinists should be apparent. Financial and other pressures currently exist to declare some people as so debilitated that they should no longer be considered persons whose lives are worth living. Historians have traced the acceptability of euthanasia in America and Europe to acceptance of evolutionary thought. "The most pivotal turning point in the early history of the euthanasia movement was the coming of Darwinism to America" [70]. The acceptability of killing as a legitimate response to certain patients reveals similar attitudes today. In spite of being illegal and against their code of ethics, hundreds of nurses in Belgium have given lethal injections to patients, often without their consent [71]. Doctors are practicing infanticide in Belgium and other countries without waiting for it to become legal [72]. While difficult ethical decisions must be made with, and about, people at the end of life, claiming their lives are not worth living has a dangerous precedent.

#### 5. Survival of the fittest

The often unspoken motivation underlying selection in bioethics today is the notion of survival of the fittest. Unlike the Nazi era, a totalitarian regime is not forcing its view of fitness on society. Instead, fitness is being determined more individually. The idea is the same, however: when people fall below the accepted standards for life, their existence can be terminated. When the parents pursuing a cloned baby were asked about the risk of abnormalities in their potential child, they responded, "We have discussed this with Dr. Zavos and, if there are abnormalities, we will abort" [73].

Prenatal genetic diagnosis can help people prepare for problems with the pregnancy, delivery, or health of their child. But in a culture where survival of the fittest is the accepted paradigm, it also creates ethical dilemmas surrounding who should survive after the diagnosis. Richard Dawkins, a leading British populariser of Darwinism and evolutionary ethics, claims the problem lies in the traditional ethic that we seek to hang on to. "The argument of this book is that we, and all other animals, are machines created by our genes. ... Much as we might wish to believe otherwise, universal love and the welfare of the species as a whole are concepts which simply do not make evolutionary sense" [74]. Hence, he too advocates the killing of certain members of our species.

### Inherent human dignity

The concern here is not that proponents of social Darwinism will usher in another Nazi-like regime. Similarities between then and now are not suggested to claim that Nazi practices are morally equivalent to what social Darwinists today endorse. There are important differences, but there are also crucial similarities. We must recognize where these beliefs led in the past, and understand why this might have happened. Regardless of what Darwinian and neo-Darwinian evolution may tell us about physical development, the history of social Darwinism reveals that its approach to ethics is destructive for humanity.

Health care systems around the world are facing huge economic pressures, just as occurred at the beginning of the twentieth century in Germany. These pressures can lead to policies that remove rights from certain humans. Their deaths may allow access to cells, tissues, or organs that will help others. The resources needed to keep them alive might be better spent elsewhere. Besides, it may become difficult to see any value in their continued existence. Economic scarcity becomes dangerous when coupled with a view that the good of society trumps the rights of individuals.

A ballast is required to counterbalance such pressures. The inherent dignity of all humans, no matter how disabled or at what stage of development, provides such a ballast. Any sliding scale of human dignity inevitably leads to undignified treatment of those humans who don't meet the standard of the day.

Underlying social Darwinism, and its more recent formulations, is a devaluing of human life. When humans are viewed as simply part of the continuum of animal life, and as having no inherent value, their worth is estimated on a curve. Their rights become arbitrary based on their estimated fitness and potential contribution. Combined with the notion of survival of the fittest, this ethics turns destructive. As was noted in 1949 when the Nazi doctors were convicted in the Nuremburg trials:

"All destructiveness ultimately leads to self-destruction; the fate of the SS and of Nazi Germany is an eloquent example. ... The beginnings at first were merely a subtle shift in emphasis in the basic attitude of the physicians. It started with the acceptance of the attitude, basic in the euthanasia movement, that there is such a thing as life not worthy to be lived. This attitude in its early stages concerned itself merely with the severely and chronically sick" [75].

Such a shift is again occurring today within bioethics. A search is under way for a secular ethic that will somehow defend human dignity. But beliefs about human worth go to the core of our worldviews and require that we admit discussion of all approaches, whether materialistic, philosophical or theological. Wilson, the sociobiologist, holds that we must either base our ethics on rational, naturalistic science or acknowledge that to some extent we need help and guidance from a dimension beyond the purely natural. He rejects the latter option because he refuses to accept a spiritual side to humanity. Roger Trigg, in his survey of views of human nature puts the option this way:

"If I think that humans are indeed a little lower than the angels, and may live on beyond this life, then I shall view myself differently from the person who accepts that the species *Homo sapiens *is one animal species amongst many, characterized by a particular evolutionary history. The tug between seeing humans as packages of genes, existing without purpose, and as the special creation of God is the modern version of a perennial debate amongst philosophers" [76].

## Summary

The concept of human dignity changed dramatically during the first half of the twentieth century under the influence of social Darwinism. The inherent dignity and special value of humans was rejected which permitted widespread destruction of human life during the Nazi era. Such an ethic was influenced by five tenets central to social Darwinism: that morality is relativistic, that humans do not have a unique status, that human dignity is relative, that some lives are not worth living, and that survival of the fittest is an ethical principle. Such beliefs are becoming more prevalent in bioethical discourse and have profound implications for current ethical and social issues. Without a robust adherence to the notion that all human life is dignified, and that human dignity is inherent and endowed, destruction of human life will increasingly be seen as the ethical answer to moral quandaries in medicine, nursing and biotechnology.

## Competing interests

The author(s) declares that he has no competing interests.

## Authors' contributions

The author is responsible for the entire manuscript.

## Pre-publication history

The pre-publication history for this paper can be accessed here:


